# Prevalence of Oral Deleterious Habits among children: A systematic review and meta-analysis

**DOI:** 10.1016/j.jobcr.2025.09.004

**Published:** 2025-09-20

**Authors:** Geetha Gireesh Gyra, Vijay S. Kumar, Chandrashekar Janakiram

**Affiliations:** Department of Public Health Dentistry, Amrita Vishwa Vidyapeetham, Amrita School of Dentistry, Kerala, India

**Keywords:** Children, Oral deleterious habits, Prevalence

## Abstract

**Objective:**

This systematic review and meta-analysis aim to estimate the prevalence of oral deleterious habits in children, providing essential evidence for targeted prevention and intervention strategies.

**Introduction:**

Oral habits such as thumb-sucking, lip biting, nail-biting, bruxism, mouth breathing, and tongue thrusting significantly contribute to dentofacial anomalies, which can potentially result in malocclusions. Understanding their prevalence is crucial for developing early intervention protocols to mitigate long-term oral health complications.

**Methods:**

The review protocol was registered with PROSPERO (CRD42024511134) and adhered to the PRISMA guidelines. A comprehensive literature search was conducted across multiple electronic databases, including PubMed, CINAHL, Cochrane Library, Scopus, Web of Science, and APA PsycINFO. Grey literature was identified through ProQuest, Google Scholar, and the Shodhganga database. Eligible studies were subjected to meta-analysis.

**Results:**

Out of 1211 identified records, 54 studies encompassing a total of 53,119 children aged 3–18 years were included. The pooled prevalence of oral deleterious habits was 28.9 %, with mouth breathing (21.1 %) and bruxism (19.0 %) being the most commonly reported habits. The habits were slightly more prevalent among males (29.4 %) than among females (26.9 %). In males, bruxism was the most frequent habit (19.9 %), whereas lip biting was the least common (6.1 %). Among females, bruxism was also most prevalent (17.6 %), with lip biting being the least reported (5.9 %). The prevalence was highest in children aged 6–12 years (32.1 %), followed by those aged 3–6 years (25.2 %), and lowest among adolescents (17.1 %).

**Conclusion:**

Oral deleterious habits are prevalent among children and can have adverse effects on dental and maxillofacial development. Early identification and timely intervention are critical to preventing malocclusions and reducing the burden of long-term orthodontic treatment.

## Graphical abstract

**Figure:**
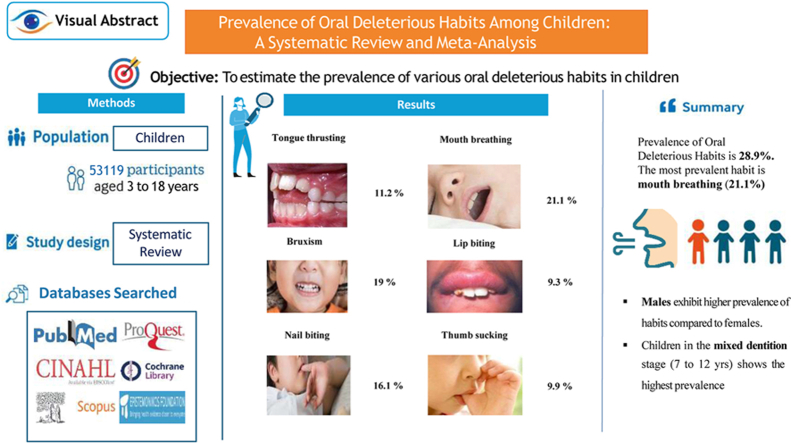

## 1 Introduction

Habits are repetitive behaviours or practices, often performed unintentionally, that become ingrained over time. In the context of oral health, certain behaviours, when persistent, can exert unbalanced functional forces on the developing dentition and associated structures. These are referred to as oral deleterious habits (ODH) and can result in significant dento-skeletal disturbances during childhood and adolescence.

ODH are associated with various oral and dental health issues, including speech difficulties, altered facial growth, malocclusion, and emotional or psychological impacts.^1^ Viggiano et al. established a clear relationship between non-nutritive sucking habits and altered occlusion.^2^ Depending on cultural practices and socioeconomic status, the prevalence and types of oral habits may vary across different communities.^3^

Common ODH observed in children include mouth breathing, thumb or finger sucking, tongue thrusting, lip biting, nail biting, and bruxism. A systematic review by Abreu et al. reported a notably high prevalence of mouth breathing among children, at 55 %.^4^ However, other reviews on similar themes have had limitations. For instance, Silva et al. conducted a brief review on deleterious oral habits, but lacked adherence to systematic review protocols, thus limiting their validity.^5^ Gois Santos et al. conducted a systematic review investigating the link between deleterious oral habits and asthma,^6^ while Castro Cunha et al. proposed to examine the associations between deleterious sucking habits and otitis media.^7^ Despite these individual efforts, there remains no comprehensive systematic review focused solely on estimating the overall prevalence of ODH in children globally.

Numerous primary studies have reported the prevalence of specific habits across different countries and demographic groups. For example, Orimadegun et al. estimated the prevalence of nutritive sucking habits among Nigerian children at 45.2 %.^8^ Vishnoi et al. identified tongue thrusting as the most prevalent oral habit in their study, affecting 28.8 % of children.^9^ The prevalence of nail-biting was reported as 22.3 % by Ghanizadeh et al.,^10^ while Dhull et al. noted that lip-biting was common among preschoolers (13.4 %).^11^

Geographic variability has also been well-documented. Prevalence rates also differ significantly by region, ranging from 13.1 % in Ile-Ife, Nigeria^12^ to 85 % in Albania,^13^ with other locations such as Bitola, Macedonia (35.39 %)^14^ and Bhubaneswar, India (36 %),^15^ falling in between, suggesting significant regional variation in both the type and frequency of habits. Such variability underscores the need for a systematic synthesis of epidemiological data to identify global trends, patterns, and high-risk populations.

The methodology, age group sampled, diagnostic criteria used, and definitions applied varied considerably across these studies, making direct comparisons and pooled estimates difficult. These discrepancies necessitate conducting a standardised systematic review to synthesise available data and accurately estimate the global burden of ODH in children. This systematic review aims to estimate the pooled global prevalence of oral deleterious habits in children aged 3–18 years, by synthesising point prevalence data from previously published observational studies.

### 1.1 Review question

What is the prevalence of oral deleterious habits among children of 3–18 years old?

## 2 Methods

The proposed systematic review was conducted using JBI methodology for systematic reviews of prevalence studies.^16^ The review title has been registered in PROSPERO with registration number CRD4202451113. This systematic review agreed with the Preferred Reporting Items for Systematic Reviews and Meta-Analyses (PRISMA) 2020 statement.^17^

**Inclusion criteria:** This systematic review was designed to comprehensively evaluate the prevalence of oral deleterious habits (ODH) among the paediatric population. Studies were included if they focused on children aged 3–18 years, without restrictions based on geographical location, ethnicity, or setting (community or institutional). The review considered studies that investigated any of the commonly reported ODH, including mouth breathing, thumb or finger sucking, tongue thrusting, nail biting, lip biting, and bruxism. Only studies that explicitly reported prevalence estimates for one or more of these habits were included. The review excluded studies that involved children with physical or mental disabilities, as well as those with chronic medical conditions such as neurological disorders, congenital syndromes, or systemic illnesses, which may independently influence oral motor function and behavioural patterns. This exclusion was aimed at avoiding confounding factors that could distort the prevalence estimates in the general paediatric population.

### 2.1 Types of studies

In terms of study design, the types included both analytical observational studies, such as prospective and retrospective cohort studies, case-control studies, and analytical cross-sectional studies, as well as descriptive cross-sectional studies that presented baseline prevalence data. Additionally, experimental studies that provided baseline prevalence information before intervention were also included, provided the data were relevant and clearly extractable. Studies were required to present original data, and only peer-reviewed publications were considered for inclusion to maintain methodological rigour and data quality.

### 2.2 Search strategy

A comprehensive search strategy was employed in three sequential steps to identify relevant studies for this systematic review. The first step involved an initial limited search of the PubMed database, using a combination of words and phrases found in the title and abstract fields. Keywords were also searched using MeSH (Medical Subject Headings) terms to ensure broad and relevant coverage of the topic. Following this, all identified keywords and indexing terms were adapted to suit each database included in the review. The second step involved tailoring this search strategy for each specific database to ensure consistent and sensitive retrieval of literature. Additionally, the reference lists of all selected full-text studies were manually screened to identify further potentially eligible articles. Reviews published in the English language from the database's inception to the present were included. The databases searched for this review included PubMed, Cochrane Database of Systematic Reviews, Scopus, Web of Science, Embase, CINAHL (EBSCOhost), Epistemonikos, ProQuest Dissertations and Theses, and Shodhganga Dissertations and Theses. Google Scholar was manually searched, focusing on the first 20 pages of search results based on the PubMed strategy to capture grey literature and other secondary sources. In addition to databases, review registers such as the Cochrane Database of Systematic Reviews, PROSPERO, and the JBI Systematic Review Register were also searched to identify ongoing or recently completed reviews relevant to the topic.^18^^,^^19^

### 2.3 Study selection

Following the database search, all retrieved citations were imported into Rayyan software, and duplicate records were systematically removed^20^ A preliminary pilot test was conducted to ensure consistency in the screening process. Titles and abstracts of the remaining studies were then independently reviewed by two researchers to determine their relevance according to the inclusion criteria. Studies that appeared to meet the criteria or required further evaluation were retrieved in full. The full texts of these articles were then independently assessed by two reviewers (GG and KR) for eligibility. At this stage, studies that did not meet the inclusion criteria were excluded, and the specific reasons for their exclusion were documented to ensure transparency. Any disagreements between the two reviewers were resolved through discussion, and if consensus was not reached, a third reviewer (CJ) was consulted. The entire study selection process, including the number of records identified, screened, excluded, and included, along with reasons for exclusion at the full-text stage, was detailed in the final systematic review and presented using a PRISMA flow diagram.

### 2.4 Data extraction

Two independent reviewers were responsible for extracting data from the studies included in the review, using a predefined spreadsheet program (Microsoft Excel®). The data extraction process focused on key study details, which include the study ID, the country where the study was conducted, the year of the study, the age group of the participants, the sample size (total number of participants), and the confidence interval for the pooled estimate. In addition to these, the total number of participants exhibiting deleterious habits was recorded, along with the prevalence of each specific habit. The goal was to gather a comprehensive understanding of the prevalence of various oral deleterious habits across different populations. Any disagreements between the two reviewers regarding the data extraction process were resolved through discussion with a third reviewer (CJ). This process ensured that all extracted data was accurate, consistent, and reliable. Once the data were finalised, it was used for quantitative synthesis to assess the prevalence and patterns of these behaviours.

### 2.5 Assessment of methodological quality

The methodological quality of eligible studies was assessed by two independent reviewers (GG and KR) using standardised critical appraisal instruments from JBI for observational studies. The assessment focused on evaluating the studies at the outcome level to ensure the reliability and robustness of the findings. The JBI methodological quality assessment tool consists of nine questions; each aimed at determining the rigour and validity of the studies. For each question, the studies were rated as follows: “Yes” (scoring 3 points), “Unclear” (scoring 2 points), or “No” (scoring 1 point). The overall score for each study was then calculated based on these ratings.^21^ Disagreements between the two reviewers regarding the scoring of any study were resolved through discussion, with a third reviewer (CJ) involved if necessary. The results of the critical appraisal were summarized in both narrative form and tabular format, which provided a clear overview of the quality of the studies reviewed. For each question, the reviewers indicated what constituted acceptable levels of information to receive a “Yes,” “Unclear”, or “No” response. Regardless of the results from the methodological quality assessment, all studies were included in the data extraction and synthesis process.

### 2.6 Data synthesis

The studies included in the review were pooled into a statistical meta-analysis using the JBI SUMARI software.^22^ The effect sizes were expressed as proportions, with 95 % confidence intervals (CIs) around the summary estimate. Statistical analyses were conducted using a random effects model, with a double arcsine transformation applied to ensure the stability of the proportion estimates, especially for extreme prevalence rates.

Subgroup analyses were performed where sufficient data were available to investigate potential differences between specific groups or study characteristics, and text was added where appropriate to highlight key findings. Heterogeneity across the studies was assessed using standard statistical tests, specifically the chi-squared and I-squared tests, to determine whether variability in results was due to true differences between studies or random chance.

The pooled prevalence of oral deleterious habits was calculated with a 95 % Confidence Interval (CI), based on the total sample size and the number of children affected by these habits. All study data, including the quantitative results from each study, were presented in a table format to ensure transparency of the findings and to allow for easy comparison across the studies included in the review. This approach ensured that the results were both comprehensive and statistically rigorous, providing a clear picture of the prevalence of oral deleterious habits among children.

## 3 Results

### 3.1 Study selection

**Fig. 1:**
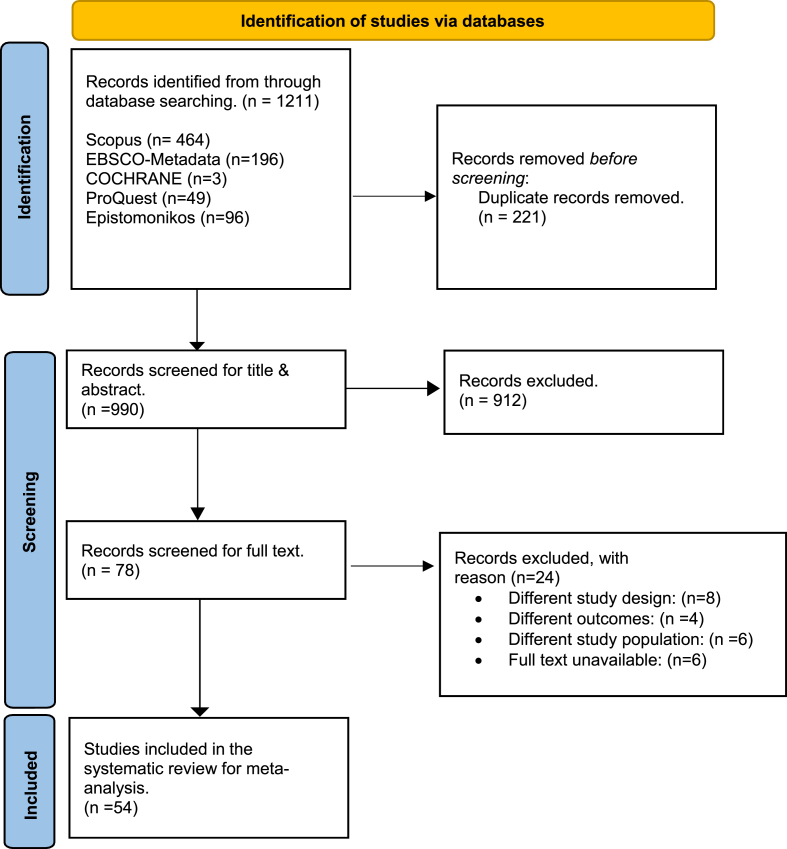
Prisma flow chart.

The PRISMA flow chart of the study selection process is shown in Figure 1. This study began by conducting an extensive search across multiple academic databases, including PubMed, Cochrane Database of Systematic Reviews, Scopus, Web of Science, Embase, CINAHL (EBSCOhost), Epistemonikos, ProQuest Dissertations and Theses, and Shodhganga Dissertations and Theses. Initially, a total of 1211 articles were retrieved from these databases.

After eliminating potential duplicates, 990 articles remained for initial screening. A total of 912 articles were excluded because they did not meet the inclusion criteria based on their titles or abstracts. The remaining 78 articles were selected for full-text screening, where more in-depth assessments were made regarding their eligibility.

During the full-text screening phase, 24 articles were excluded for various reasons. The reasons for exclusions included: Eight studies with different study designs, four studies that focused on different outcomes than the ones required for this review, six studies that involved a different age group than the target population of children aged 3 to 18, and six studies for which full-text access was not available. As a result, 54 articles were finally deemed eligible and included in the quantitative synthesis of the review.

The 54 included studies were then carefully analysed to assess the prevalence of oral deleterious habits among children. The study selection process was documented and illustrated using a PRISMA flow diagram to ensure transparency and reproducibility. By applying this rigorous selection process, the final set of 54 articles provided a comprehensive and methodologically sound basis for investigating the prevalence of harmful oral habits across different populations of children.

### 3.2 Study characteristics

The characteristics of the individual studies included in this systematic review are summarized in Table 1. A total of 54 prevalence studies were reviewed, with publication years ranging from 1991 to 2023. These studies were identified through comprehensive searches across multiple databases. Notably, more than half of the studies were conducted in Brazil, underscoring the country's extensive research focus on paediatric oral health and its commitment to understanding the burden of deleterious oral habits in children.

The cumulative sample across all included studies was 54,419 children, with ages ranging from 3 to 18 years. This broad age range enables the examination of oral habit prevalence across various stages of dental development—deciduous, mixed, and permanent dentition. Most of the studies employed a cross-sectional design. Only one study adopted a case-control design, reflecting the predominance of observational methodologies in this domain.

#appsec2Sample sizes varied considerably, from as few as 151 participants to as many as 6,389, representing diverse population demographics. In terms of methodology, the majority of studies used both questionnaires and clinical examinations to identify oral habits, allowing for triangulation of findings. However, 16 studies relied solely on questionnaires, which may have limited the objectivity and accuracy of habit identification due to potential biases in self-reporting or parental reporting.

Despite the strengths of this review, several methodological limitations were identified. Three studies had an inappropriate sample frame, which may affect the representativeness of their findings. Eight studies lacked appropriate sampling methods, potentially introducing selection bias. Two studies had inadequate sample sizes, limiting statistical power. In one study, the subjects and study setting were not sufficiently described, impeding contextual understanding. Additionally, two studies did not adequately cover the identified sample in their data analysis, while four studies lacked robust statistical analyses. Furthermore, in some cases, the condition (oral habit) was not measured in a standardised and reliable way across participants. Eight studies also reported inadequate response rates, which could compromise the validity of their prevalence estimates.

These methodological variations and quality issues underscore the importance of interpreting the pooled prevalence estimates with caution. Nonetheless, the inclusion of such a wide range of studies enriches the understanding of global trends and provides valuable insights into the epidemiology of deleterious oral habits in children.

### 3.3 Assessment of methodological quality

The methodological quality of the individual studies included in this systematic review is presented in Table 2. The studies were assessed using the JBI checklist for prevalence studies, which consists of nine critical appraisal questions. Each study was scored based on its response to these questions, with a scoring system of Yes (3 points), Unclear (2 points), and No (1 point). The maximum possible score for each study is 27, and the results varied across the studies.

The highest score of 27 was achieved by 25 studies, indicating a strong methodological quality in these studies. On the other hand, the lowest score was nine, observed in one study, suggesting significant methodological limitations. Notably, 22 studies achieved a perfect score of 9 out of 9 appraisal criteria, highlighting their adherence to methodological rigour. Seventeen studies met 8 out of the 9 criteria, showing that they were essentially sound in their design and execution.

However, several issues were identified in certain studies. In three studies, the sample frame was deemed inappropriate for addressing the target population, which may affect the generalizability of the findings. Additionally, eight studies had issues with the sampling process, where the participants were not selected in an appropriate manner, potentially leading to sampling biases. In two studies, the sample size was found to be inadequate, which could limit the reliability of the prevalence estimates.

Furthermore, one study did not provide a sufficient description of the subjects and settings, which could hinder the understanding and reproducibility of the research. In two of the studies, the data analysis did not provide adequate coverage of the identified sample. Four studies lacked appropriate statistical analysis, which is crucial for ensuring the robustness and validity of the conclusions drawn from the data. Inadequate response rate was found in eight of the studies, which could introduce non-response bias and affect the representativeness of the sample. These methodological limitations should be considered when interpreting the results of the review, as they may influence the accuracy and generalizability of the findings.

### 3.4 Data synthesis

The meta-analysis of 54 studies revealed significant insights into the prevalence of oral deleterious habits (ODH) among children, with an overall prevalence rate of 28.9 % (95 % CI: 24.6–33.4) (Figure 2). This suggests that nearly a third of children exhibit oral deleterious habits. Among these, mouth breathing emerged as the most prevalent habit, with a prevalence of 21.1 % (95 % CI: 13.1–30.4) (Figure 2a). Following mouth breathing, bruxism—grinding or clenching of teeth—was found in 19 % of children (95 % CI: 14.9–23.5) (Figure 2f).

Other significant oral habits included nail-biting, with a prevalence of 16.1 % (95 % CI: 9.4–24.3) (Figure 2e), and tongue thrusting (11.2 %, 95 % CI: 4.3–20.6) (Figure 2c). Thumb or finger sucking was observed in 9.9 % of the children (95 % CI: 5.5–15.3) (Figure 2b), while lip biting was the least prevalent habit, occurring in just 9.3 % of children (95 % CI: 3.5–17.5) (Figure 2d).

Analysis based on gender revealed that males exhibited a higher prevalence of oral deleterious habits compared to females. The overall prevalence in males was 29.4 % (95 % CI: 24.8–34.3) (Figure 3Figure 3aFigure 3aa), while in females it was 26.9 % (95 % CI: 22.4–31.6) (Figure 3b). Among males, bruxism was the most common habit, affecting 19.9 % of children (95 % CI: 14.8–25.7) (Figure 9a), while lip biting was the least common, with a prevalence of 6.1 % (95 % CI: 1.4–6.0) (Figure 7a). Similarly, for females, bruxism was also the most prevalent habit, affecting 17.6 % of children (95 % CI: 12.6–23.2) (Figure 9b), and lip biting remained the least common habit, affecting 5.9 % of females (95 % CI: 1.6–12.5) (Figure 7b).

Age-wise analysis revealed a marked variation in the prevalence of oral deleterious habits. The 6–12 years age group, which corresponds to the mixed dentition period, exhibited the highest prevalence of ODH at 32.1 % (95 % CI: 23.8–41.0) (Figure 10b). Children aged 3–6 years also had a notable prevalence of 25.2 % (95 % CI: 19.2–31.6) (Figure 10a). Conversely, adolescents aged 12–18 years had the lowest prevalence of ODH, with a rate of 17.1 % (95 % CI: 9.9–25.9) (Figure 10c).

## 4 Discussion

Various oral and orofacial disorders significantly impact children, potentially affecting their daily functioning, well-being, and quality of life (QoL). Non-nutritive oral habits, such as thumb sucking and tongue thrusting, exert unbalanced forces on the developing dentition, which can result in malocclusion.^23^ Oral deleterious habits are a primary contributing factor to dental malalignment in children. These habits can lead to aesthetic concerns, potentially resulting in social discomfort and reduced self-confidence.

This systematic review aims to quantify the prevalence of various deleterious oral habits among children. The prevalence reported by Anila et al. (72.7 %) is considerably higher than that observed in the present review.^24^ Conversely, Krishnappa et al. documented a significantly lower prevalence of 6 % among children aged 10–16 years in Karnataka.^1^ The current review reports a higher prevalence of the habits among males than females, which is similar to the finding by Vishnoi et al. (58.55 % vs. 56.25 %).^9^ The prevalence of oral deleterious habits among preschool children (3–6 years) in a study by Dhull et al. is 36 %, which is higher than the current review.^11^

In a study by Masutomi et al., a mouth breathing prevalence of 20 % among children was reported, which is similar to the current review.^25^ A systematic review by Ziyi Zhao et al. reported that children with mouth breathing exhibited backwards and downward rotation of the mandible and maxilla, along with a steep occlusal plane.^26^ According to Souki et al., mouth breathers commonly exhibit anterior open bite, posterior crossbite, and Class II malocclusion.^27^

A review by Renuka Devi et al. reports a prevalence of 35–55 % of tongue thrusting habit among children of 3–5 years old which is much higher than the finding of the present review.^28^ According to Tarvade et al., tongue thrusting contributes to the proclination and in some cases, flaring of the upper anterior teeth, resulting in an increased overjet. It may also result in the retroclination or proclination of the lower anterior teeth, as well as the development of anterior or posterior open bite.^29^

“Traisman and Traisman reported that 45.6 % of American children under the age of four engaged in thumb sucking habit, which is much higher than in the present review.^30^ According to Ahmed ZN et al., the habit of finger or thumb sucking may contribute to the development of an asymmetrical anterior open bite.^31^ A diverse variety of approaches has been used to help children with an appliance to interfere with the habit, application of an aversive taste to the digit or behaviour modification techniques.^32^

Zeilinski et al. report a global prevalence of bruxism is 22.2 % which is in accordance with the current review.^33^ Insana et al. found a high prevalence of sleep bruxism among preschool children (36.8 %).^34^ Bruxism assessment showed a positive association with genetics, quality of life aspects (school and emotional functions and overuse of screen-time), mother anxiety and family conformation, diet, alteration in sleep behaviours and architecture, and sleep breathing disorders.^35^

Foster et al. estimated the prevalence of nail-biting habit among pre-school children in the USA as 23 %, which is in contrast to the present review (16.1 %).^36^ Anxiety was the most common antecedent, according to self-monitoring recordings. It was determined that the most effective way to stop the undesirable habit was to increase self-awareness. During the course of treatment, Transcendental Meditation and deep muscular relaxation were employed as systematic desensitisation strategies.^37^ Dhull et al. reported lip biting as the most prevalent habit (13.4 %) among preschool children, whereas in the present review, it was the least prevalent habit (9.3 %).^15^ A functional appliance (F.A.) can be utilised for the management of lip-sucking and lip-biting habits. Its use facilitates the cessation of these deleterious oral habits while contributing to the correction of overjet and overbite, ultimately enhancing the facial profile.^38^

Early intervention aimed at addressing the underlying causes of these habits is essential in preventing the onset or progression of malocclusion. In cases where malocclusion has already developed, early orthodontic treatment is recommended to support proper skeletal and dental development.^39^ Parents should be educated on the various types of deleterious oral habits, their etiology, and the role of stress in their development. Additionally, they should be guided on evidence-based strategies for preventing, managing, and implementing home-based interventions for these habits.^40^

## 5 Strengths and limitations

This is the comprehensive systematic review of deleterious oral habits. Including 54 studies, exceeding the number in previous reviews, enabled a thorough evaluation of the prevalence of oral habits in children. The pooled prevalence of each specific habit was calculated, and subgroup analyses were conducted to assess gender- and age-specific trends. This provides valuable insights into the timing of interventions, potentially preventing the development of malocclusion.

However, there are several limitations. The prevalence of certain habits, such as bruxism, may be underestimated as many studies relied on parental reporting. Additionally, the pooled prevalence of bruxism did not differentiate between awake and sleep bruxism, which may have led to some habits or associated factors being overlooked. Many studies did not report prevalence data separately for males and females, limiting the generalizability of the subgroup analyses. Finally, using a random-effects model in the meta-analysis may have influenced variability in the results.

## 6 Conclusion

The findings highlight the significant prevalence of deleterious oral habits in children, with variations based on age, gender, and geographic location. Based on the review, it was found that the prevalence of ODH among 3-18-year-olds was high across various geographical locations. The most prevalent habit is mouth breathing, which highlights the potential importance of addressing breathing patterns as part of oral health interventions. The least prevalent is lip biting. Males were found to have a higher prevalence of ODH compared to females. Bruxism was found to be the most prevalent ODH among both males and females, underscoring its commonality as a damaging oral habit. These gender-based differences may suggest that while some habits are universally prevalent, the degree of their impact varies across genders. The highest prevalence of ODH is among preschool children. This period is critical as the transition from primary to permanent teeth occurs, and habits formed during this phase may have lasting effects on dental and oral health. The prevalence of ODH is less frequently observed among adolescents. This finding suggests that as children grow older, they may be less likely to engage in such habits, potentially due to increased awareness of oral health or developmental changes in oral and facial structures. Early identification and intervention are crucial in preventing the long-term consequences of these habits, the most significant being malocclusion.

## Patients/guardians consent

Not Applicable as this is a systematic review.

## Plain text summary

This study examined the prevalence of everyday harmful oral habits among children, including thumb-sucking, nail-biting, teeth grinding (bruxism), and mouth breathing. These habits can interfere with the normal growth of teeth and jaws, often leading to issues such as crooked teeth or bite problems. Understanding how many children are affected helps in creating ways to prevent or manage these habits early. This research focused on children aged 3–18 years and did not include studies involving children with disabilities or long-term medical conditions. After reviewing a large number of studies, the researchers included 54 that met their criteria, encompassing data from over 53,000 children. It was found that around 29 out of every 100 children had at least one harmful oral habit. Mouth breathing was the most common (affecting about 21 %), followed by teeth grinding (affecting 19 %). Boys were slightly more likely to have these habits than girls. These habits were seen most often in children aged 6–12 years and were less common in teenagers. The study shows that a significant number of children have habits that can affect their dental and facial development. Spotting and addressing these habits early can help prevent future dental issues and reduce the need for braces or other costly treatments.

## Ethical clearance

Not Applicable as this is a systematic review.

## Consent form

As it is a secondary research (systematic review), we don't require consent as data is collected from published secondary sources.

## Source of funding

None.

## Declaration of competing interest

Authors declare none.

## Figures and Tables

**Table:** 

Search	Query	Records retrieved
#1	“Mouth breathing" [All Fields] OR “Tongue thrusting" [All Fields] OR “fingersucking" [MeSH Terms] OR “Lip biting" [All Fields] OR “nail biting" [MeSH Terms] OR “bruxism" [MeSH Terms] OR “sleep bruxism" [MeSH Terms]	7036
#2	“Prevalence" [MeSH Terms] OR “epidemiology" [MeSH Terms] OR “cross sectional studies" [MeSH Terms]	7,97,761
#3	“child" [MeSH Terms] OR “disabled children" [MeSH Terms] OR “dental care for children" [MeSH Terms] OR “infant" [MeSH Terms] OR “Children" [All Fields]	34,27,907
	((#1) AND (#2)) AND (#3)	403

**Table:** 

Search	Query	Result
#1	“Prevalence"	50875
#2	MeSH descriptor: [Prevalence] explode all trees	7385
#3	“cross-sectional studies"	10902
#4	MeSH descriptor: [Cross-Sectional Studies] explode all trees	9825
#5	incidence	158213
#6	MeSH descriptor: [Incidence] explode all trees	14311
#7	epidemiology	88772
#8	“cohort -studies"	14923
#9	#1 OR #2 OR #3 OR #4 OR #5 OR #6 OR #7 OR #8	269555
#10	“ fingersucking"	7
#11	MeSH descriptor: [Fingersucking] explode all trees	10
#12	“ mouthbreathing"	219
#13	MeSH descriptor: [Mouth Breathing] explode all trees	57
#14	“nail biting"	46
#15	MeSH descriptor: [Nail Biting] explode all trees	17
#16	bruxism	517
#17	MeSH descriptor: [Bruxism] explode all trees	207
#19	MeSH descriptor: [Sleep Bruxism] explode all trees	98
#20	#10 OR #11 OR #12 OR #13 OR #14 OR #15 OR #16 OR #17 OR #18 OR #19	791
#21	“child, orphaned"	52
#22	“only, child"	557
#23	“child, preschool"	40343
#24	adolescent	164416
#25	MeSH descriptor: [Adolescent] explode all trees	136503
#26	“adolescent, health"	2312
#27	#21 OR #22 OR #23 OR #24 OR #25 OR #26	188222
#28	#9 AND #20 AND #27	3

**Table:** 

Search	Query	Result
#1	“Tongue thrusting” OR “thumb sucking"OR “Lip biting” OR “Nail Biting” OR “Finger sucking” OR “Nail Biting"OR “Sleep Bruxism “OR “Bruxism"	4867
#2	“Prevalence” OR “Epidemiology” OR “epidemiology” OR “Cross-Sectional Studies"	11,05,339
#3	“Child” OR “Disabled Children” OR “Dental Care for Children” OR “Infant” OR “Children”	13,74,867
	#1 AND #2 AND #3	49

**Table:** 

Search	Query	Result
#1	“Tongue thrusting” OR “thumb sucking"OR “Lip biting” OR “Nail Biting” OR “Finger sucking” OR “Nail Biting"OR “Sleep Bruxism “OR “Bruxism"	22,586
#2	“Prevalence” OR “Epidemiology” OR “epidemiology” OR “Cross-Sectional Studies"	80,79,775
#3	“Child” OR “Disabled Children” OR “Dental Care for Children” OR “Infant” OR “Children”	89,53,207
	#1 AND #2 AND #3	464

**Table:** 

Search	Query	Result
#1	“Tongue thrusting” OR “thumb sucking"OR “Lip biting” OR “Nail Biting” OR “Finger sucking” OR “Nail Biting"OR “Sleep Bruxism “OR “Bruxism"	1772
#2	“Prevalence” OR “Epidemiology” OR “epidemiology” OR “Cross-Sectional Studies"	92,87,834
#3	“Child” OR “Disabled Children” OR “Dental Care for Children” OR “Infant” OR “Children”	38,11,558
	#1 AND #2 AND #3	196

**Table:** 

Search	Query	Result
#1	“Tongue thrusting” OR “thumb sucking"OR “Lip biting” OR “Nail Biting” OR “Finger sucking” OR “Nail Biting"OR “Sleep Bruxism “OR “Bruxism"	12,709
#2	“Prevalence” OR “Epidemiology” OR “epidemiology” OR “Cross-Sectional Studies"	719
#3	“Child” OR “Disabled Children” OR “Dental Care for Children” OR “Infant” OR “Children”	558
	#1 AND #2 AND #3	96

**Fig. 2:**
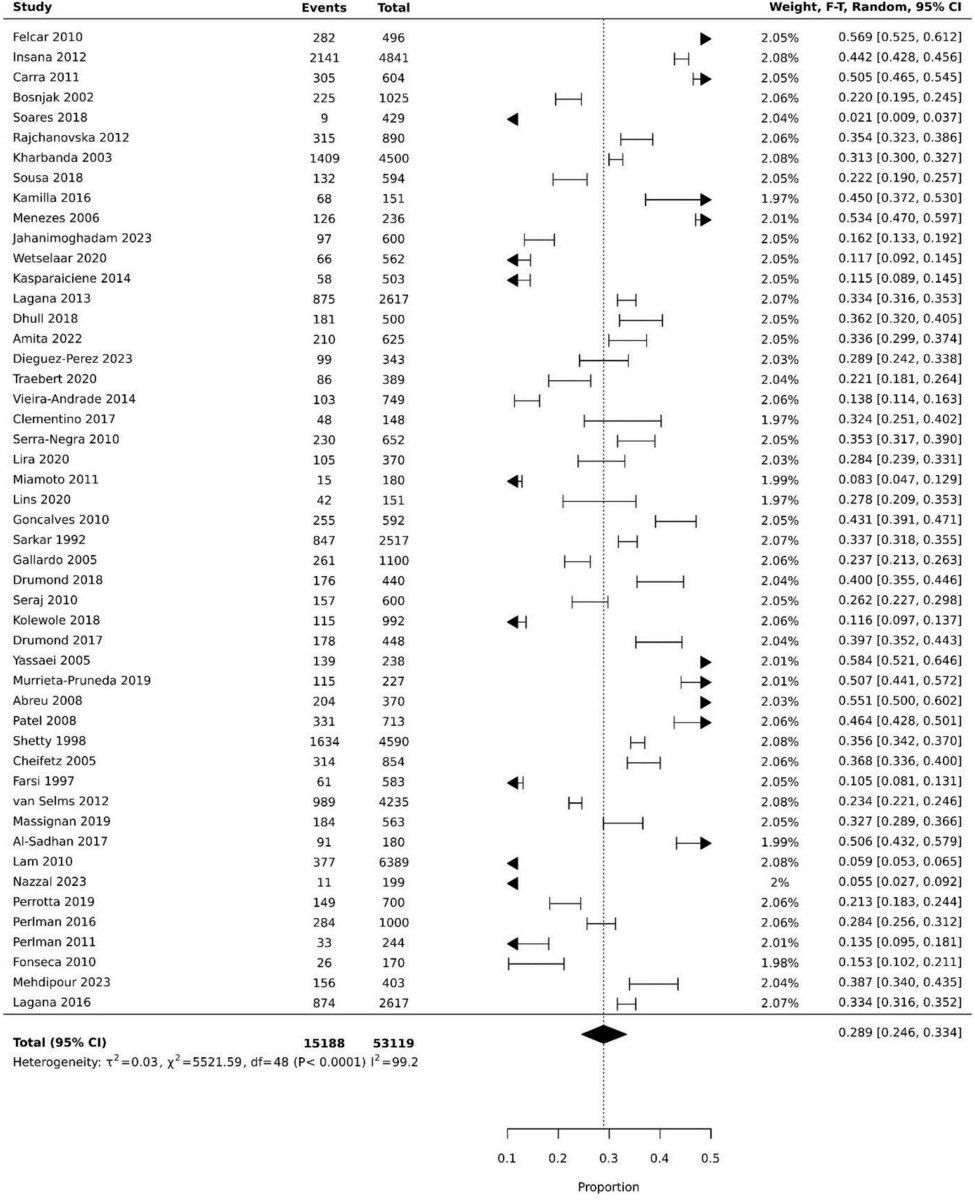
Prevalence of oral deleterious habits combined.

**Fig. 2a:**
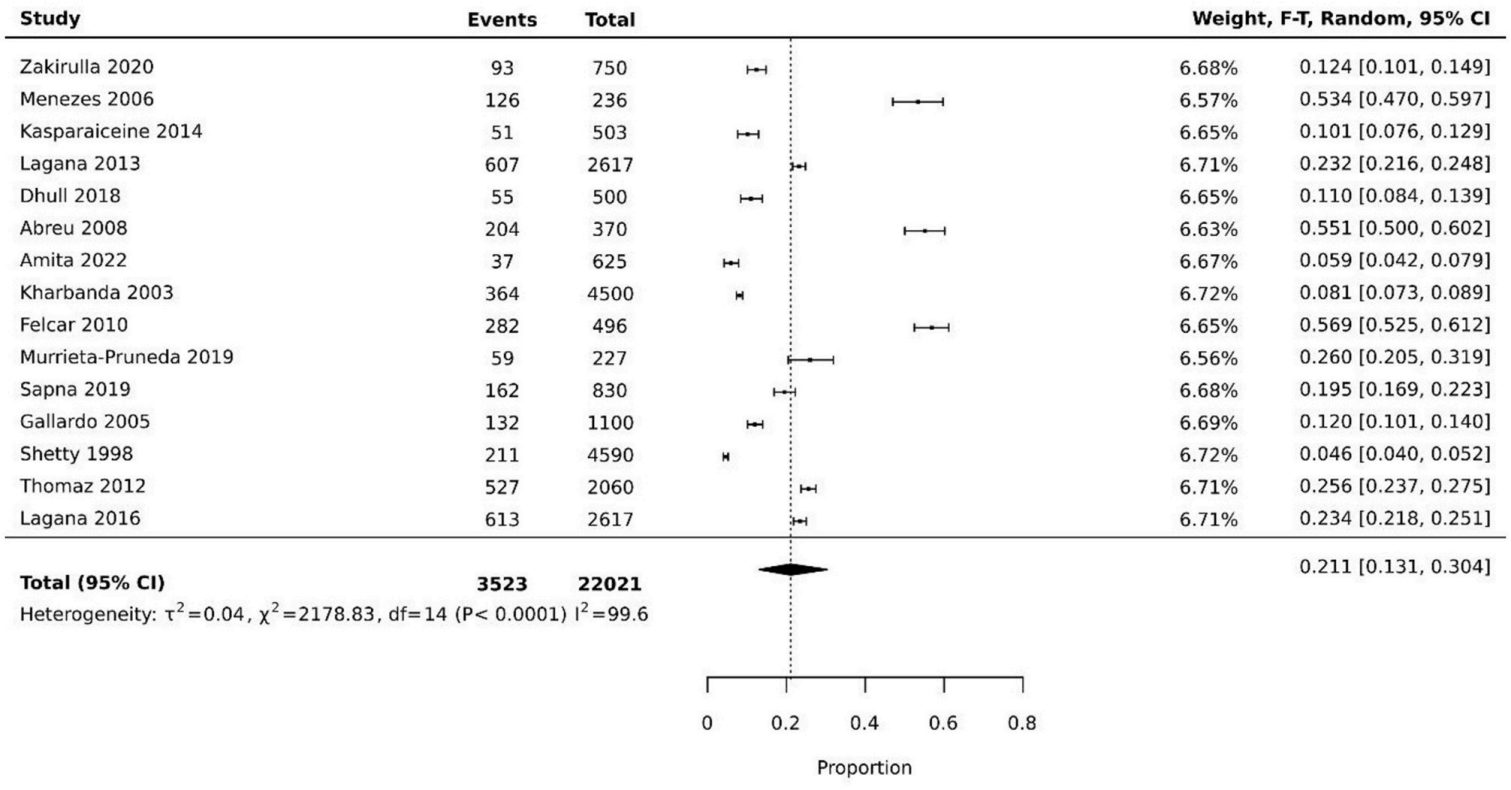
Prevalence of oral deleterious habits-mouth breathing.

**Fig. 2b:**
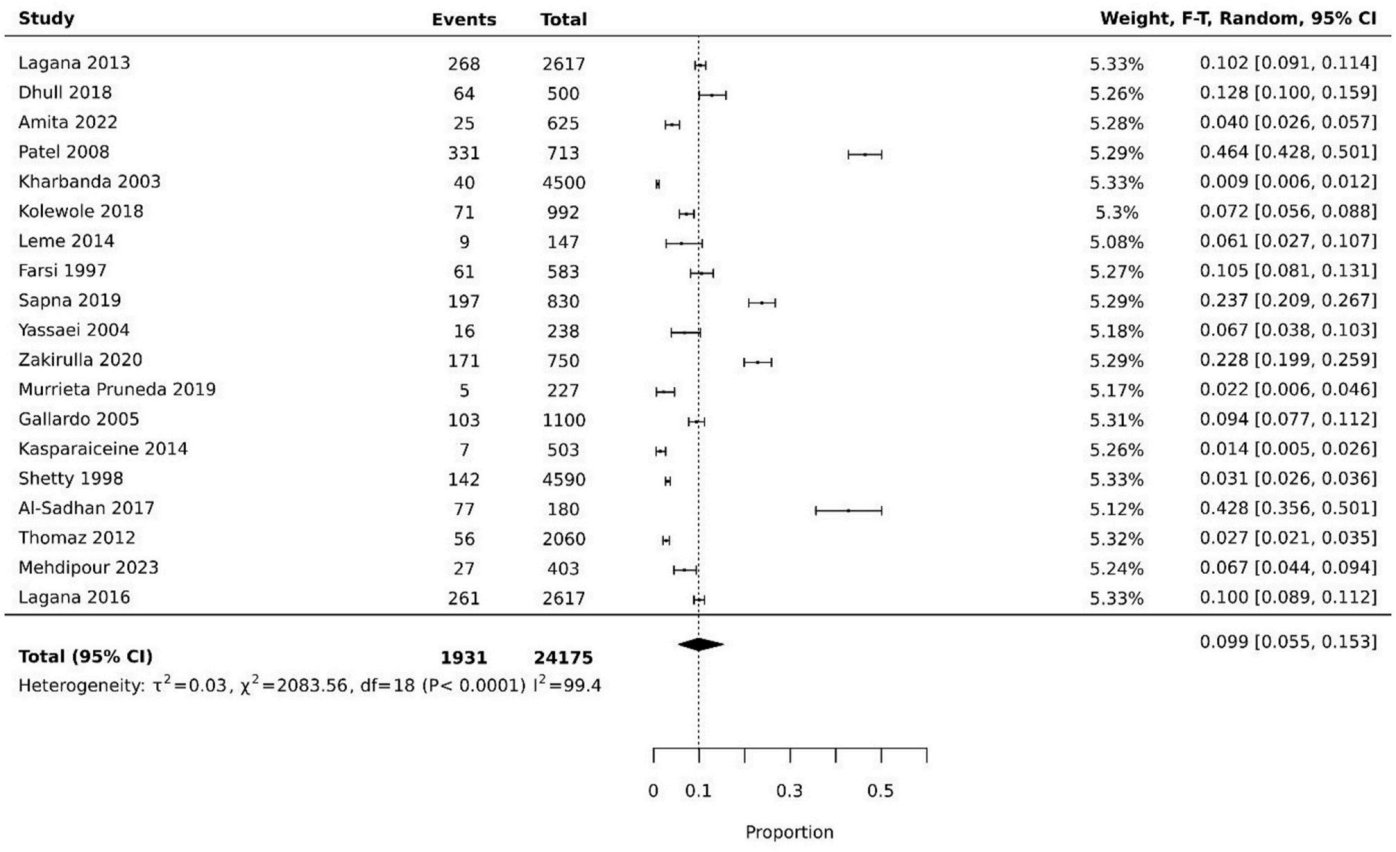
Prevalence of oral deleterious habits-thumb/ finger sucking.

**Fig. 2c:**
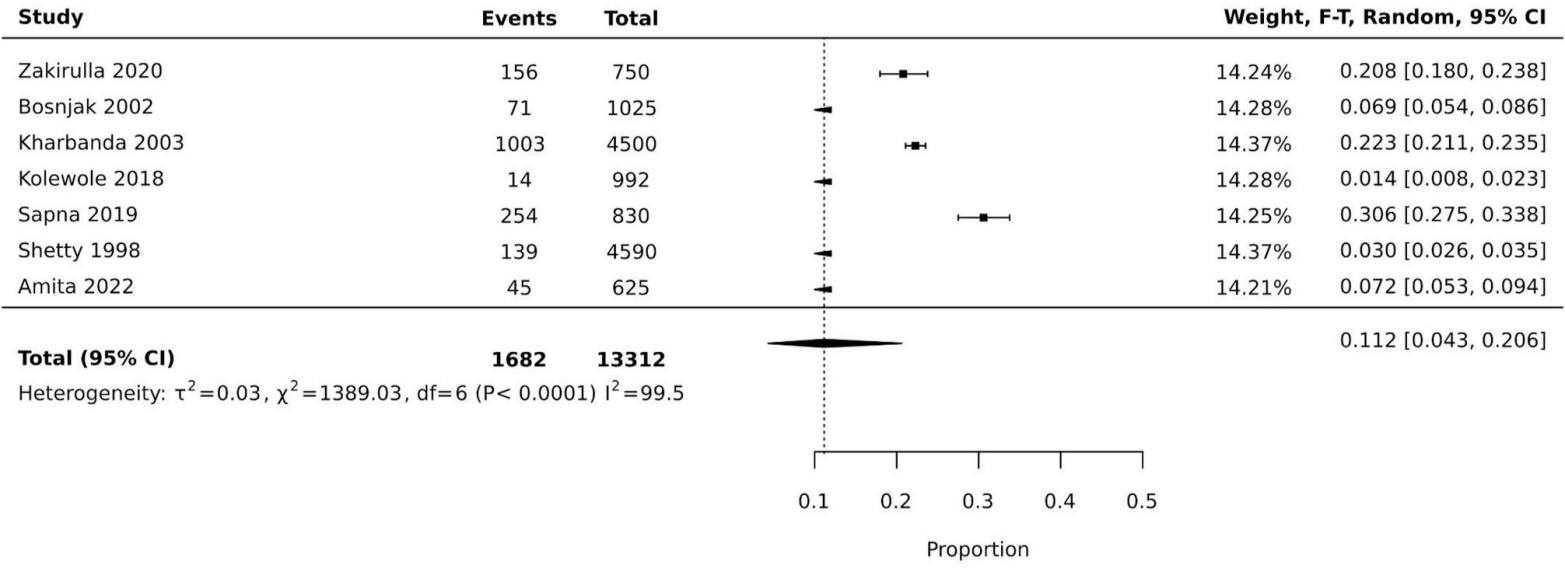
Prevalence of oral deleterious habits-tongue thrusting.

**Fig. 2d:**
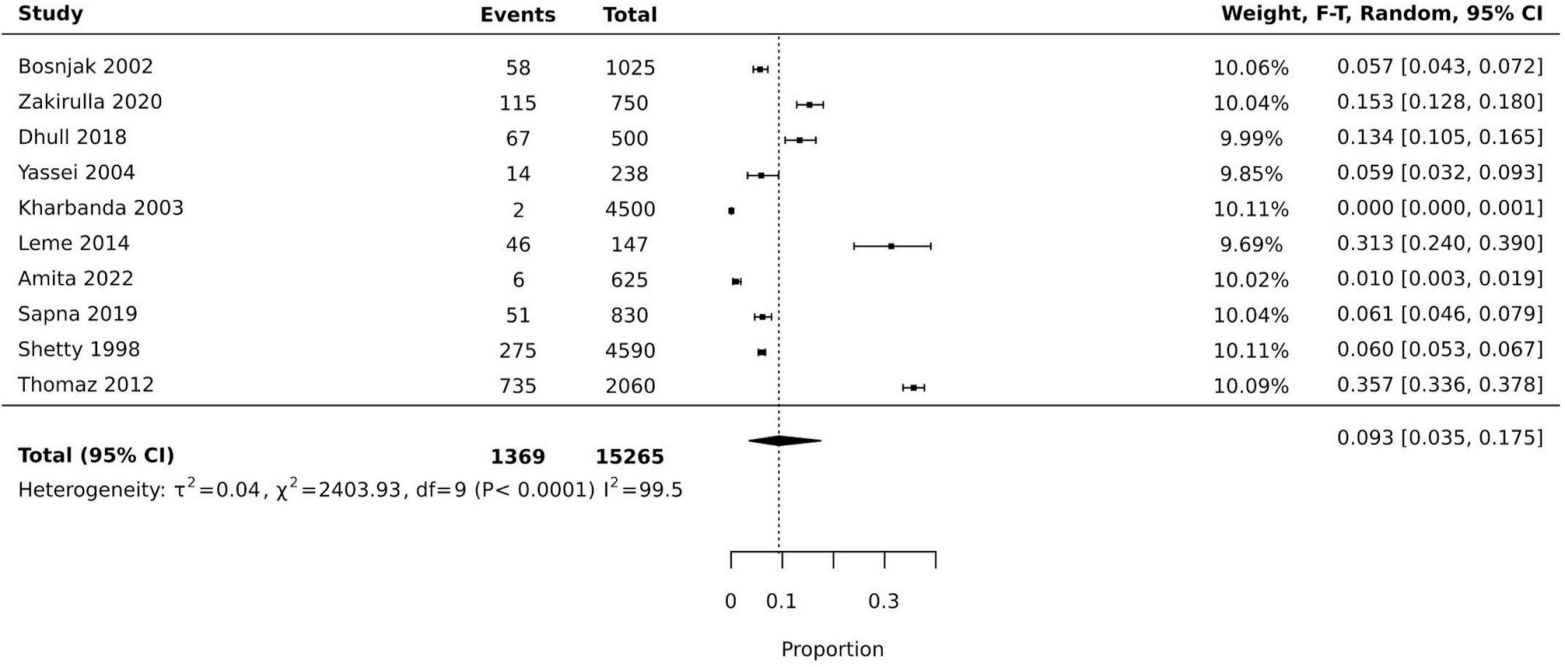
Prevalence of oral deleterious habits-lip biting.

**Fig. 2e:**
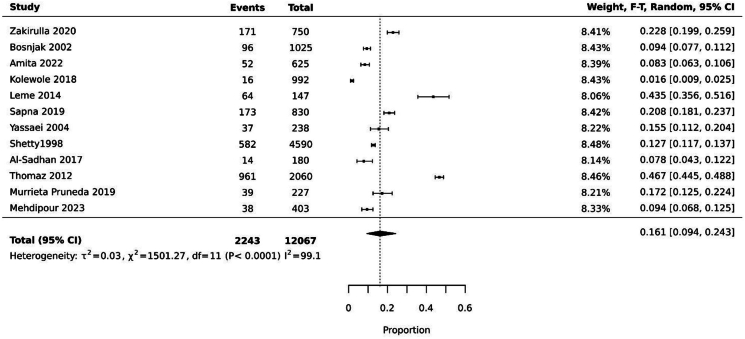
Prevalence of oral deleterious habits-nail biting.

**Fig. 2f:**
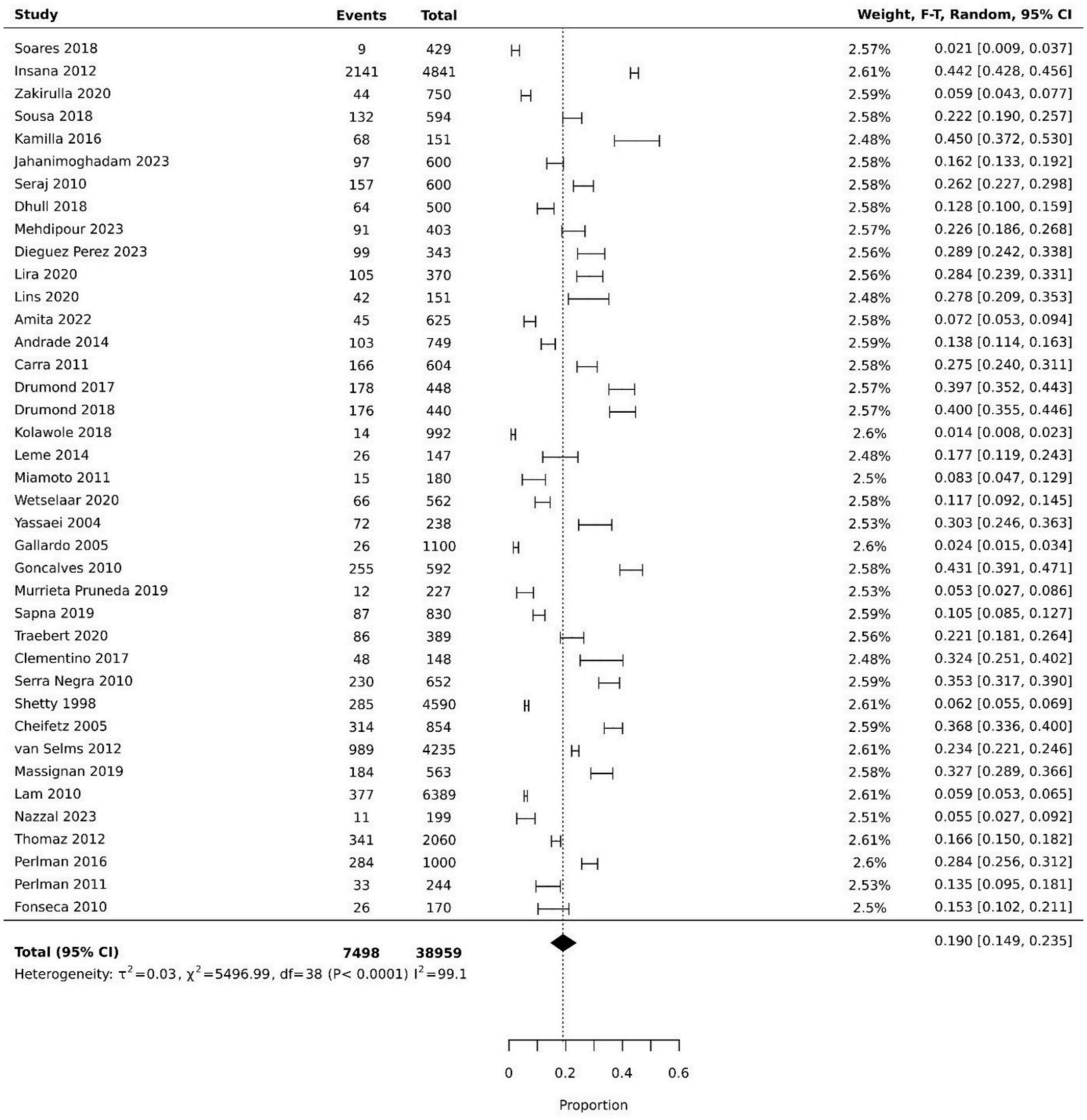
Prevalence of oral deleterious habits-bruxism.

**Fig. 3a:**
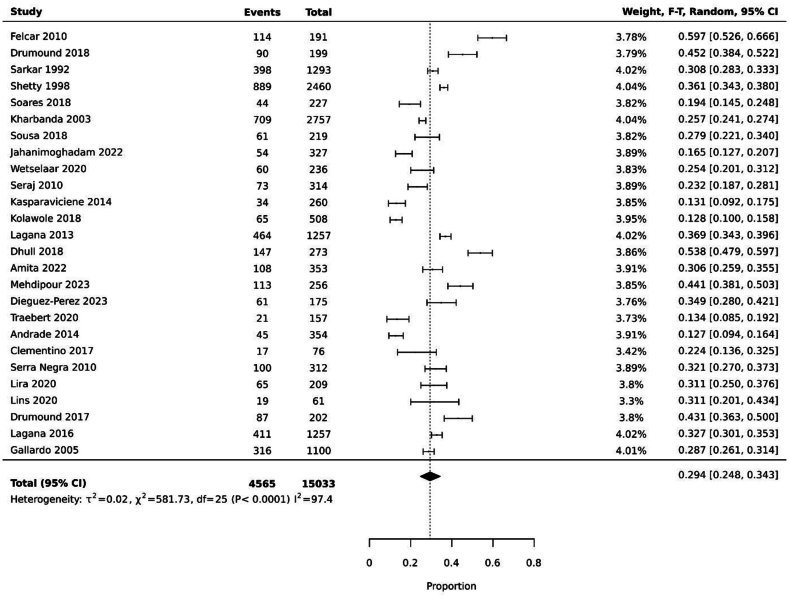
Prevalence of oral deleterious habits-Males.

**Fig. 3b:**
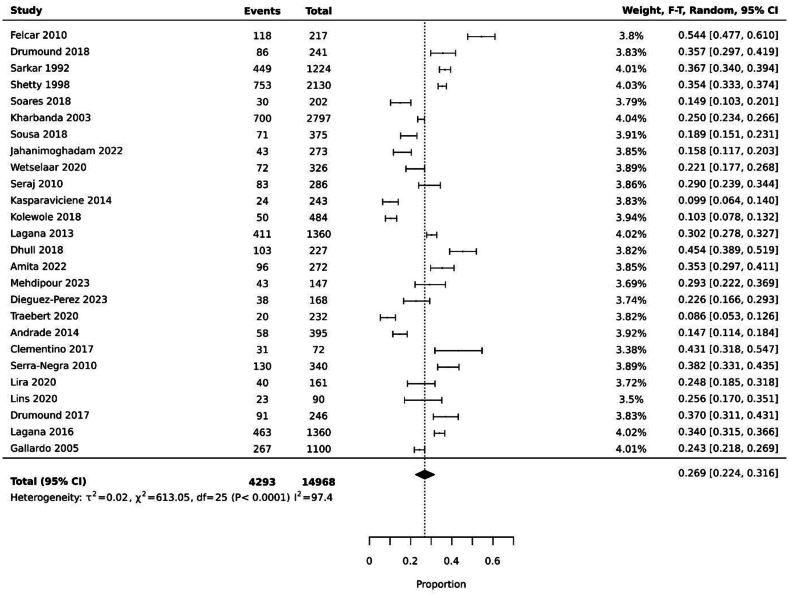
Prevalence of oral deleterious habits-Females.

**Fig. 4a:**
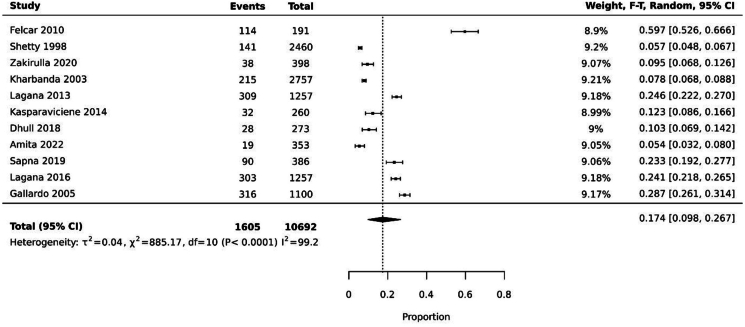
Prevalence of mouth breathing-Males.

**Fig. 4b:**
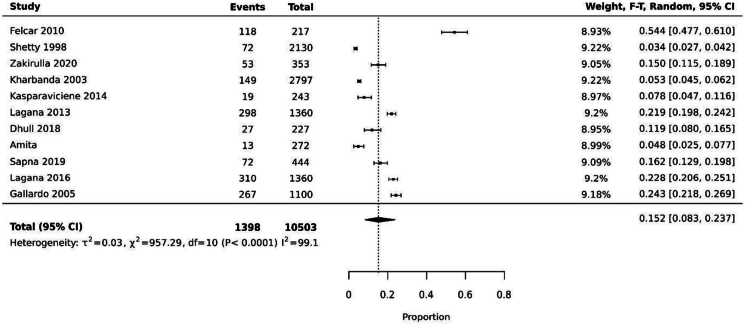
Prevalence of mouth breathing- Females.

**Fig. 5a:**
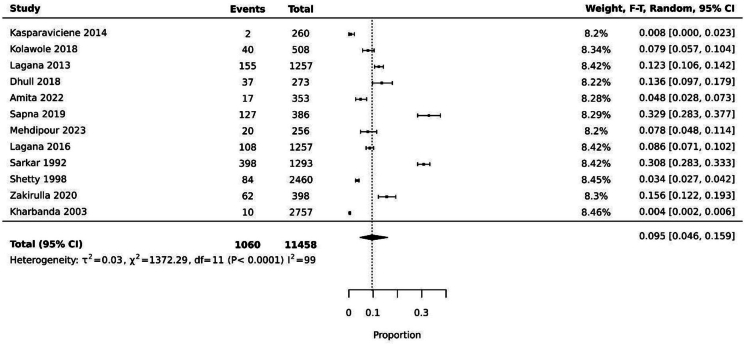
Prevalence of thumb or finger sucking-Males.

**Fig. 5b:**
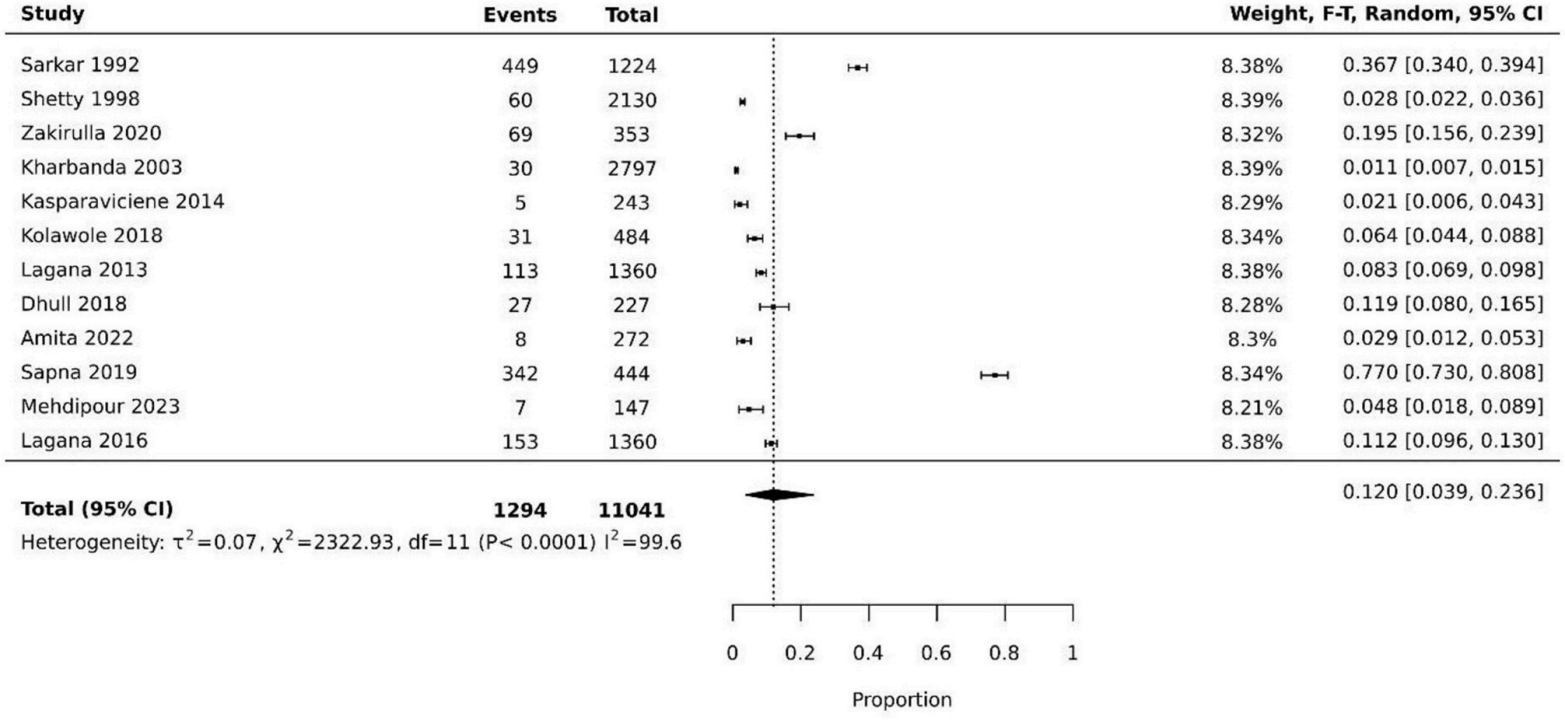
Prevalence of thumb or finger sucking-Females.

**Fig. 6a:**
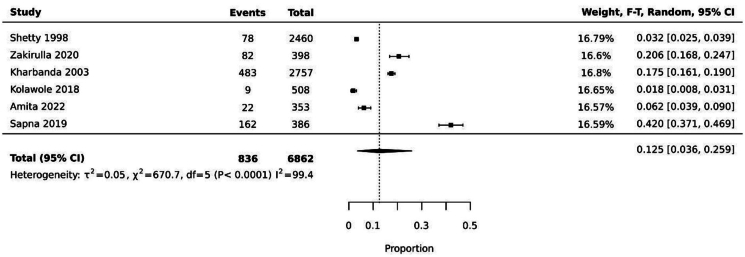
Prevalence of tongue thrusting-Males.

**Fig. 6b:**
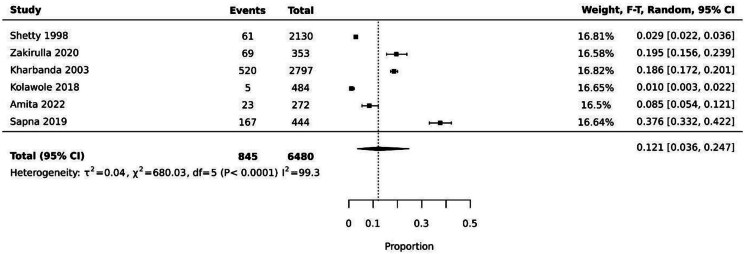
Prevalence of tongue thrusting-Females.

**Fig. 7a:**
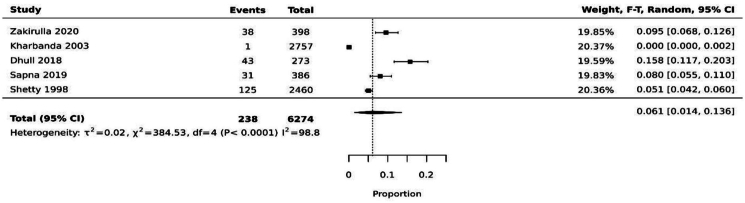
Prevalence of lip biting-Males.

**Fig. 7b:**
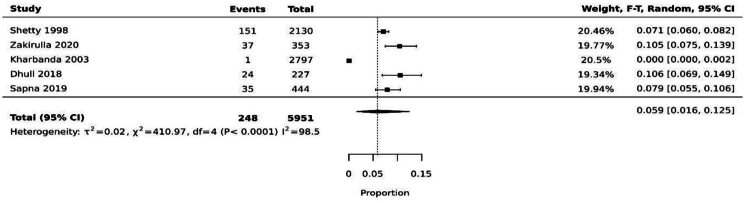
Prevalence of lip biting-Females.

**Fig. 8a:**
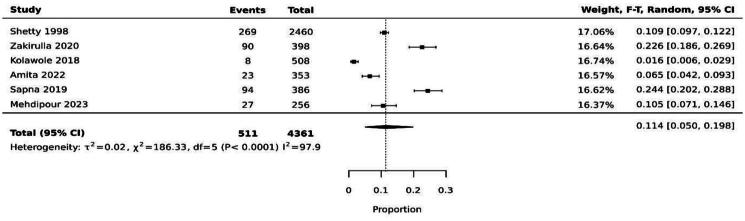
Prevalence of nail biting-males.

**Fig. 8b:**
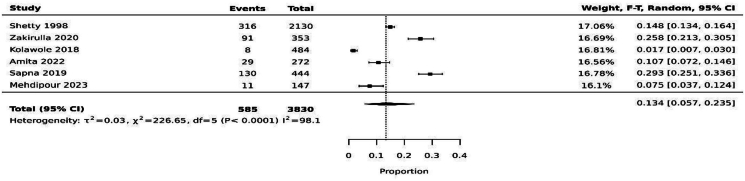
Prevalence of nail biting-Females.

**Fig. 9a:**
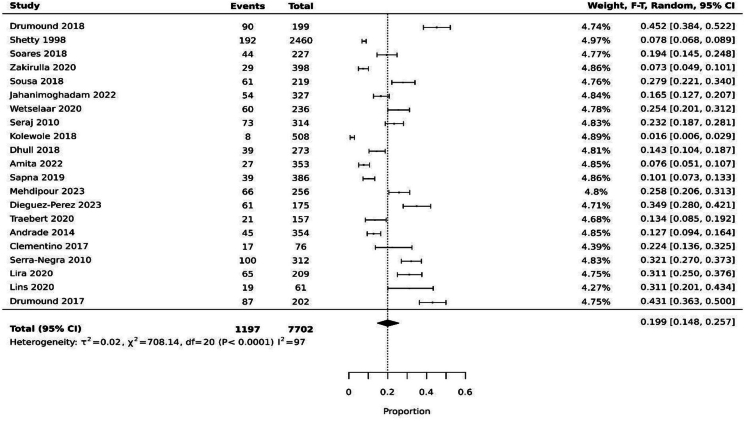
Prevalence of bruxism-Males.

**Fig. 9b:**
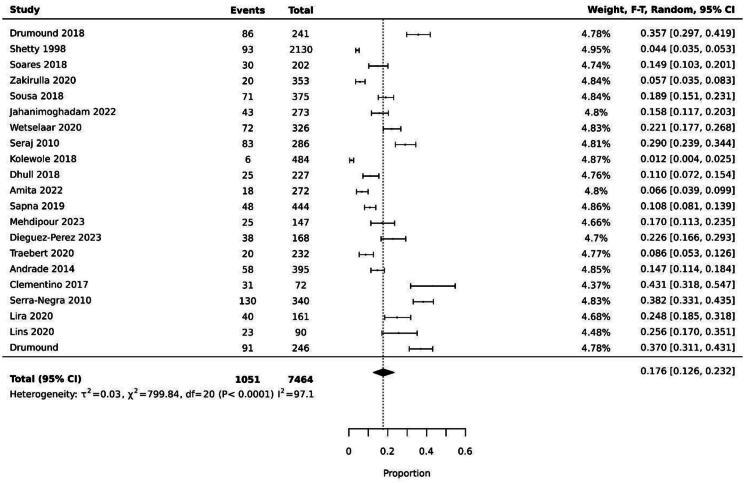
Prevalence of bruxism-Females.

**Fig. 10a:**
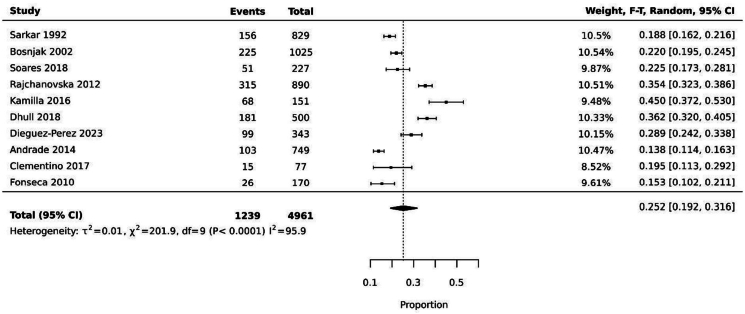
Prevalence of oral deleterious habits among children of 3–6 years.

**Fig. 10b:**
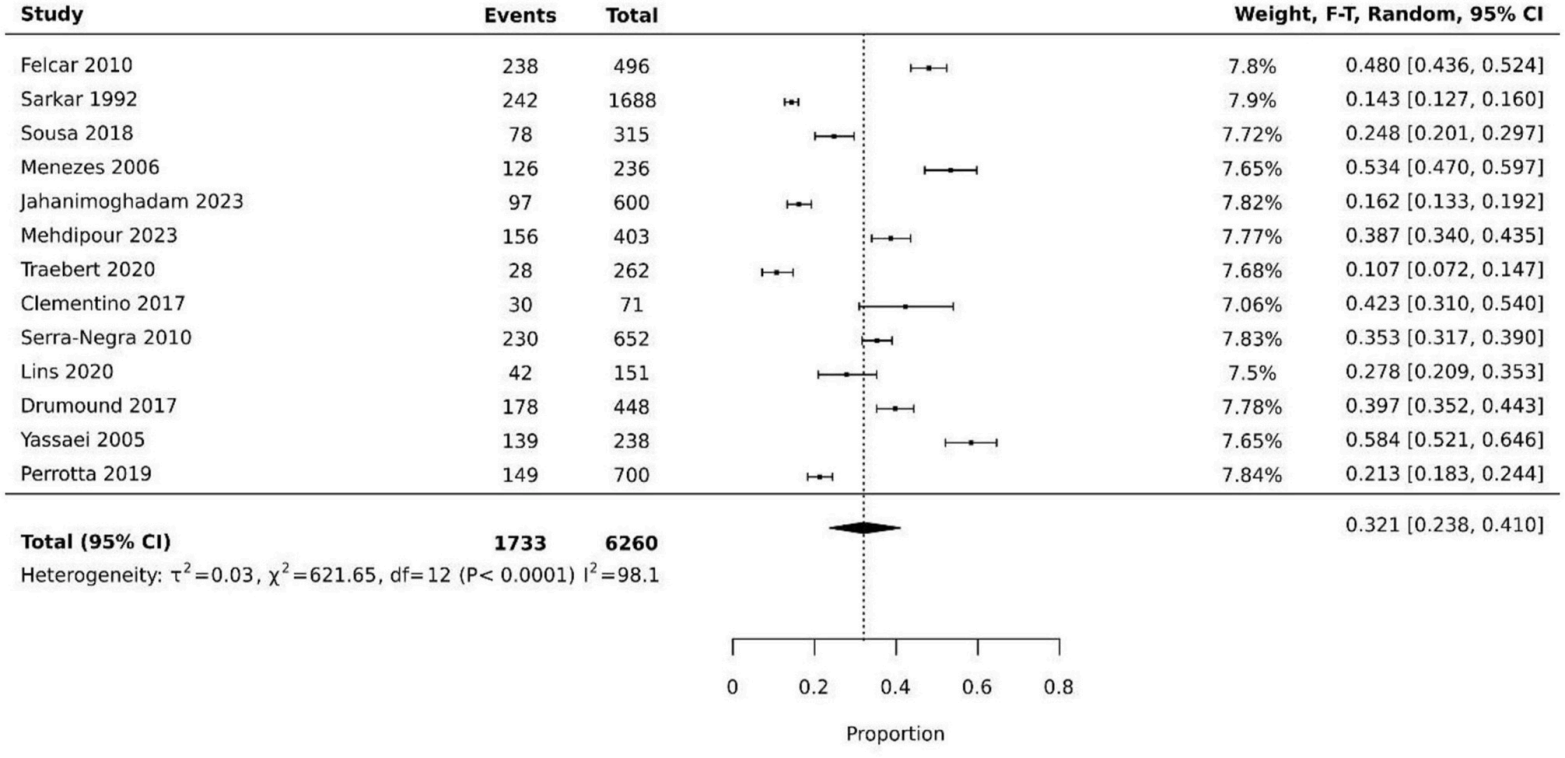
Prevalence of oral deleterious habits among children of 6–12 years.

**Fig. 10c:**
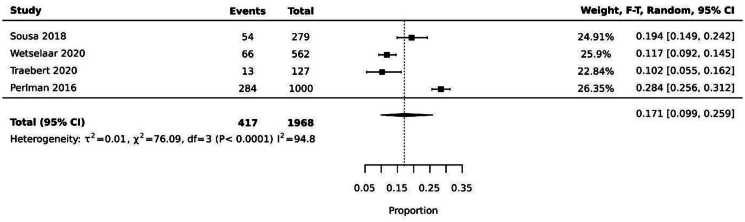
Prevalence of oral deleterious habits among children of 12–18 years.

**Table 1:** Characteristics of included studies

Serial No.	Study ID	Country of study	Year of study	Age group	Type of study	Total no. of participants	Assessment of oral deleterious habits	Type of Oral Deleterious Habits
1	Felcar 2010	Portugal	2008	6–9 years	Cross-sectional study	496	Questionnaire, Clinical examination	MB
2	Gallardo 2005	Spain	2003	4–11 years	Cross-sectional study	1100	Questionnaire, Clinical examination	MB, FS, PS, Atypical swallow, bruxism
3	Leme 2014	Brazil	2008	8–14 years	Cross-sectional study	147	Questionnaire, Clinical examination	Pen/pencil biting, TB, LB, NB, PS, FS, PS, Grinding
4	Insana 2012	USA	2012	2.5–8.6 years	Cross-sectional study	4841	Questionnaire, Clinical examination	Bruxism
5	Prado 2023	Brazil	2018	12–19 years	Cross-sectional study	403	Questionnaire, Clinical examination	Bruxism
6	Carra 2011	Canada	2011	7–17 years	Cross-sectional study	604	Questionnaire, Clinical examination	Bruxism
7	Drumound 2018	Brazil	2018	8–10 years	Cross-sectional study	440	Questionnaire, Clinical examination	Bruxism
8	Bosnjak 2002	Croatia	2002	6–11 years.	Cross-sectional study	1025	Questionnaire	NB, OB, TT, LB, non-nutritive sucking
9	Soares 2018	Brazil	2017	2–5 years	Cross-sectional study	429	Questionnaire, Clinical examination	Bruxism
10	Rajchanovska 2012	Macedonia	2009	3–5 years	Cross-sectional study	890	Questionnaire, Clinical examination	NB, FS
11	Zakirulla 2020	Saudi Arabia	2017	7–13 years	Cross-sectional study	750	Questionnaire, Clinical examination	TS, TT, MB, LB, NB, Bruxism
12	Kharbanda 2003	India	1991	5–13 years	Cross-sectional study	4500	Questionnaire, Clinical examination	TT, MB, TS
13	Sousa 2018	Brazil	2013	11–14 years	Cross-sectional study	594	Questionnaire, Clinical examination	Bruxism
14	Kamilla 2016	Brazil	2015	3–5 years	Cross-sectional study	151	Questionnaire	Bruxism
15	Menezes 2006	Brazil	2005	8–10 years	Cross-sectional study	236	Questionnaire, Clinical examination	MB
16	Jahanimoghadam 2023	Iran	2021	6–12 years	Cross-sectional study	600	Questionnaire	Bruxism
17	Wetselaar 2020	Netherland	2017	17 years	Cross-sectional study	562	Questionnaire	Bruxism
18	Seraj 2010	Iran	2007–2008	4–12 years	Cross-sectional study	600	Questionnaire	Bruxism
19	Kasparaiciene 2014	Lithuania	2014	5–7 years	Cross-sectional study	503	Questionnaire, Clinical examination	MB, DS, infantile swallow
20	Kolawole 2018	Nigeria	2014	6–12 years	Cross-sectional study	992	Questionnaire, Clinical examination	DS, TT, LS, LB, NB, OB, bruxism
21	Laganà 2013	Italy	2009–2010	7–15 years	Cross-sectional study	2617	Questionnaire, Clinical examination	FS, MB, PS, atypical swallow, low tongue position, lip interposition
22	Dhull 2018	India	2018	3–5 years	Cross-sectional study	500	Questionnaire, Clinical examination	LB, TS, MB, bruxism
23	Amita 2022	Nepal	2017	3–7 years	Cross-sectional study	625	Questionnaire, Clinical examination	NB, DS, LB, MB, TT, PS, bruxism, self-destructive habits
24	Sapna 2019	Nepal	2017	6–13 years	Cross-sectional study	830	Questionnaire, Clinical examination	TT, OB, FS, MB, PS, LS, NB, LB, NB, bruxism, self -destructive habits
25	Mehdipour 2023	Iran	2021	6–12 years	Cross-sectional study	403	Questionnaire, Clinical examination	FS, NB, non-nutritive sucking habit, infantile swallow, bruxism
26	Diéguez-Pérez 2023	Spain	2015	3–5 years	Cross-sectional study	343	Questionnaire, Clinical examination	Bruxism
27	Traebert 2020	Brazil	2010	10–15 years	Cross-sectional study	389	Questionnaire, Clinical examination	Bruxism
28	Vieira-Andrade 2014	Brazil	2010	3–5 years	Cross-sectional study	749	Questionnaire, Clinical examination	Bruxism
29	Clementino 2017	Brazil	2015–2016	3–12 years	Cross-sectional study	148	Questionnaire	Bruxism
30	Serra-Negra 2010	Brazil	2006	7–10 years	Cross-sectional study	652	Questionnaire	Bruxism
31	Lira 2020	Brazil	2018	2–6 years	Cross-sectional study	370	Questionnaire, Clinical examination	Bruxism
32	Miamoto 2011	Brazil	2011		Cross-sectional study	180	Questionnaire, Clinical examination	Sleep bruxism
33	Drumond 2017	Brazil	2017	8–11 years	Cross-sectional study	448	Questionnaire	Sleep bruxism
34	Lins 2020	Brazil	2018	6–10 years	Cross-sectional study	151	Clinical examination	Probable sleep bruxism
35	Gonçalves 2010	Brazil	2010	4–16 years	Cross-sectional study	592	Questionnaire, Clinical examination	Bruxism
36	Yassaei 2005	Iran	2000	7–11 years	Case-control study	238	Questionnaire	TS, FS, NB, TS, LB, sleep bruxism, bruxism events
37	Murrieta-Pruneda 2019	Mexico	2019	4–5 years	Cross-sectional study	227	Questionnaire, Clinical examination	MB, FS, LS, atypical swallow, pacifier use, onychophagia, bruxism
38	Abreu 2008	Brazil	2008	3–9 years	Cross-sectional study	370	Questionnaire, Clinical examination	MB
39	Patel 2008	UK	2008	3–6 years	Cross-sectional study	713	Questionnaire	DS
40	Sarkar 1992	India	1992	3–12 years	Cross-sectional study	2517	Questionnaire	TS
41	Shetty 1998	India	1998	3–16 years	Cross-sectional study	4590	Questionnaire, Clinical examination	MB, TT, DS, NB, PB, LB, bruxism, masochistic habits
42	Cheifetz 2005	USA	2005	1–17 years	Cross-sectional study	854	Questionnaire	Bruxism
43	Farsi 1997	Saudi Arabia	1997	3–5 years	Cross-sectional study	583	Questionnaire, Clinical examination	Dummy sucking, digit sucking
44	van Selms 2012	Netherlands	2012	12–18 years	Cross-sectional study	4235	Questionnaire	Bruxism
45	Massignan 2019	Brazil	2014–2015	8–10 years	Cross-sectional study	563	Questionnaire, Clinical examination	Sleep bruxism
46	Al-Sadhan 2017	Saudi Arabia	2017	4–12 years	Cross-sectional study	180	Questionnaire, Clinical examination	DS, NB, self-mutilating habits
47	Lam 2010	Hong Kong, China	2003–2004	5–11 years	Cross-sectional study	6389	Questionnaire	Bruxism
48	Nazzal 2023	Qatar	2021	6–16 years	Cross-sectional study	199	Questionnaire	Sleep bruxism
49	Thomaz 2012	Brazil	2004	12–15 years	Cross-sectional study	2060	Questionnaire, Clinical examination	NB, OB, MB, FS, PS, lip/cheek biting, bruxism
50	Perrotta 2019	Italy	2019	9–11 years	Cross-sectional study	700	Questionnaire	Bruxism, TT, LB
51	Perlman 2016	Israel	2016	12–18 years	Cross-sectional study	1000	Questionnaire	Bruxism
52	Perlman 2011	Israel	2011	5–12 years	Cross-sectional study	244	Questionnaire, Clinical examination	NB, chewing gum, opening bottle with teeth, pen/pencil biting, chewing ice
53	Fonseca 2010	Brazil	2007	3–6 years	Cross-sectional study	170	Questionnaire, Clinical examination	Sleep bruxism
54	Lagana 2021	Italy	2018–2019	6–12 years	Cross-sectional study	310	Questionnaire	Sleep bruxism

∗MB- Mouth Breathing ∗TS- Thumb Sucking ∗FS-Finger Sucking ∗TT-Tongue Thrusting ∗LB-Lip Biting ∗NB-Nail Biting.

∗PS-Pacifier Sucking ∗TB- Tongue Biting ∗OB-Object Biting ∗DS- Digit Sucking.

**Table 2:** Methodological Quality Assessment of included studies

SL No.	Study	1	2	3	4	5	6	7	8	9	Overall Score
1	Felcar 2010	Yes	Yes	Yes	Yes	Yes	Yes	No	Yes	Yes	25
2	Gallardo 2005	No	Yes	Yes	Yes	Yes	Unclear	Unclear	Yes	Yes	22
3	Leme 2014	Yes	Yes	Yes	Yes	Yes	Yes	Yes	Yes	No	26
4	Insana 2012	Yes	No	Yes	Yes	Yes	Yes	Yes	Yes	Yes	25
5	Prado 2023	Yes	Yes	Yes	Yes	Yes	Yes	Yes	Yes	Yes	27
6	Carra 2011	Unclear	Unclear	Yes	Yes	Yes	Unclear	Yes	Yes	Unclear	20
7	Drumound 2018	Yes	Yes	Yes	Yes	Yes	Unclear	Yes	Yes	Yes	26
8	Bosnjak 2002	Yes	Unclear	Yes	Yes	Yes	Unclear	Yes	Yes	Yes	24
9	Soares 2018	Yes	Unclear	Yes	Yes	Yes	Yes	Yes	Yes	Yes	26
10	Rajchanovska 2012	Yes	Unclear	Yes	Yes	Yes	Unclear	Unclear	Yes	Yes	22
11	Zakirulla 2020	Yes	Yes	Yes	Yes	Yes	Yes	Yes	Yes	Yes	27
12	Kharbanda 2003	Yes	Yes	Yes	Yes	Yes	Unclear	Unclear	Yes	Yes	24
13	Sousa 2018	Yes	Yes	Yes	Yes	Yes	Yes	Yes	Yes	Yes	27
14	Kamilla 2018	Yes	Yes	Yes	Yes	Yes	Yes	Yes	Yes	Yes	27
15	Menezes 2006	Yes	Yes	Yes	Yes	Yes	Yes	Yes	Yes	Yes	27
16	Jahanimoghadam 2023	Yes	Unclear	Yes	Yes	Yes	Unclear	Unclear	Yes	No	19
17	Wetselaar 2020	Yes	Yes	Yes	Yes	Yes	Yes	Yes	Yes	Yes	27
18	Seraj 2010	Yes	Unclear	Yes	Yes	Yes	Yes	Yes	Yes	Yes	25
19	Kasparaiciene 2014	Yes	Unclear	Yes	Yes	Yes	Yes	Yes	Yes	Yes	25
20	Kolawol 2018	Yes	Yes	Yes	Yes	Yes	Unclear	Unclear	Unclear	Yes	23
21	Laganà 2013	Yes	Yes	Yes	Yes	Yes	Yes	Yes	Yes	No	25
22	Dhull 2018	Yes	Yes	Yes	Yes	Yes	Unclear	Yes	Unclear	Yes	24
23	Amita 2022	Yes	Yes	Yes	Yes	Yes	Unclear	Yes	Yes	Yes	25
24	Sapna 2019	Yes	Yes	Yes	Yes	Yes	Yes	Unclear	Yes	Yes	25
25	Mehdipour 2023	Yes	Yes	Yes	Yes	Yes	Yes	Yes	Yes	Yes	27
26	Pérez 2023	Yes	Yes	Yes	Yes	Yes	Yes	Yes	Yes	Yes	27
27	Traebert 2020	Yes	Yes	Yes	Yes	Yes	No	No	Yes	Yes	22
28	Andrade 2014	Yes	Yes	Yes	Yes	Yes	Yes	Yes	Yes	Yes	27
29	Clementino 2017	Yes	Yes	Yes	Yes	Yes	Yes	Yes	Yes	Yes	27
30	Negra 2010	Yes	Yes	Yes	Yes	Yes	Yes	Yes	Yes	Yes	27
31	Lira 2020	Yes	Yes	Yes	Yes	Yes	No	No	Yes	Yes	21
32	Miamoto 2011	Yes	Yes	Yes	Yes	Yes	Unclear	Yes	Yes	Yes	25
33	Lins 2020	Yes	Yes	Yes	Yes	Yes	Yes	Yes	Yes	Yes	27
34	Gonçalves 2010	Yes	Yes	Yes	Yes	Yes	Yes	Yes	Yes	Yes	27
35	Yassaei 2005	Yes	Yes	Yes	Yes	Yes	Yes	Yes	Yes	No	25
36	Pruneda 2019	Yes	Yes	Yes	Yes	Yes	Unclear	Unclear	Yes	Yes	24
37	Drumond 2017	Yes	Yes	Yes	Yes	Yes	Yes	Yes	Yes	Yes	27
38	Sarkar 1992	Yes	Yes	Yes	Yes	Unclear	Yes	Yes	Yes	Yes	25
39	Shetty 1998	Yes	Yes	Yes	Yes	Yes	Yes	Yes	Yes	No	25
40	Abreu 2008	Yes	Yes	Yes	Yes	Yes	Yes	Yes	Yes	Yes	27
41	Patel 2008	Yes	Yes	Yes	Yes	Yes	Unclear	Yes	Yes	Yes	25
42	Farsi 1997	Yes	Yes	Yes	Yes	Yes	Yes	Yes	Yes	Yes	27
43	Chieftzetal 2005	Yes	Yes	Yes	Yes	Yes	Unclear	Unclear	Yes	Yes	24
44	van Selms 2012	Yes	Yes	Yes	Yes	Yes	Yes	Yes	Yes	Yes	27
45	Massignan 2019	Yes	Yes	Yes	Yes	Yes	Yes	Yes	Yes	Yes	27
46	Al-Sadhan 2017	Yes	Yes	No	Yes	Yes	No	No	Yes	Yes	19
47	Lam 2010	Yes	Yes	Yes	Yes	Yes	Yes	Yes	Yes	Yes	27
48	Nazzal 2023	Yes	Yes	Yes	Yes	Yes	Unclear	Unclear	Yes	No	21
49	Thomaz 2012	Yes	Yes	Yes	Yes	Yes	Yes	Yes	Yes	Yes	27
50	Perrotta 2019	Yes	Yes	Yes	Yes	Yes	Yes	Unclear	Yes	Yes	25
51	Perlman 2016	Yes	Yes	Yes	Yes	Yes	Yes	Yes	Yes	Yes	27
52	Perlman 2011	Yes	Yes	Yes	Yes	Yes	Yes	Yes	Yes	Yes	27
53	Fonseca 2010	No	No	No	No	Unclear	Unclear	Unclear	Unclear	Unclear	9
54	Lagana 2021	Yes	Yes	Yes	Yes	Yes	Yes	Yes	Unclear	Yes	26

1: Was the sample frame appropriate to address the target population? 2: Were study participants sampled in an appropriate way?.

3: Was the sample size adequate? 4: Were the study subjects and the setting described in detail? 5: Was the data analysis conducted with sufficient coverage of the identified sample?6: Were valid methods used for the identification of the condition? 7: Was the condition measured in a standard, reliable way for all participants? 8: Was there appropriate statistical analysis? 9: Was the response rate adequate, and if not, was the low response rate managed appropriately?.

Yes = 3, Unclear = 2, No = 1.

**Table 3:** Consolidated Table of Pooled Estimate

Variable	No of studies included	No of subjects with oral deleterious habits	Total sample size	I^2^	Pooled prevalence (%)	Confidence interval	Reference for figure
Mouth breathing	15	3523	22021	99.6	21.1	13.1–30.4	Figure 2a
Thumb sucking	19	1931	24175	99.4	9.9	5.5–15.3	Figure 2b
Tongue thrusting	7	1682	13312	99.4	11.2	4.3–20.6	Figure 2c
Lip biting	10	1369	15265	99.5	9.3	3.5–17.5	Figure 2d
Nail biting	12	2243	12067	99.1	16.1	9.4–24.3	Figure 2e
Bruxism	42	7498	38959	99.1	19.0	14.9–23.5	Figure 2f
Oral Deleterious Habits- Males	26	4565	15033	97.4	29.4	24.8–34.3	Figure 3a
Oral Deleterious Habits - Females	26	4293	14968	97.4	26.9	22.4–31.6	Figure 3b
Mouth breathing-Males	11	1605	10692	99.2	17.4	9.8–26.7	Figure 4a
Mouth breathing-Females	11	1398	10503	99.1	15.2	8.3–23.7	Figure 4b
Thumb/finger sucking- Males	12	1060	11458	99	9.5	4.6–15.9	Figure 5a
Thumb/finger sucking- Females	12	1294	11041	99.6	12.0	3.9–23.6	Figure 5b
Tongue thrusting- Males	6	836	6862	99.4	12.5	3.6–25.9	Figure 6a
Tongue thrusting- Females	6	845	6480	99.3	12.1	3.6–24.7	Figure 6b
Lip biting-Males	5	238	6274	98.8	6.1	1.4–13.6	Figure 7a
Lip biting-Females	5	248	5951	98.5	5.9	1.6–12.5	Figure 7b
Nail biting-Males	6	511	4361	97.9	11.4	5–19.8	Figure 8a
Nail biting-Females	6	585	3830	98.1	13.4	5.7–23.5	Figure 8b
Bruxism-Males	21	1197	7702	97	19.9	14.8–25.7	Figure 9a
Bruxism-Females	21	1051	7464	97.1	17.6	12.6–23.2	Figure 9b
Oral deleterious habits in age group 2–6 years	10	1239	4961	959	25.2	19.2–31.6	Figure 10a
Oral deleterious habits in age group 6–12 years	13	1733	6260	98.1	32.1	23.8–41.0	Figure 10b
Oral deleterious habits in age group 12–18 years	4	417	1968	94.8	17.1	9.9–25.9	Figure 10c

**Table 4:** List of Excluded Articles

Serial No.	Study citation	Reason for exclusion
1	PEREZ QUINONEZ, José Alberto; MARTINEZ, Jorge Luis; MOURE IBARRA, Miriam e PEREZ PADRON, Alejandro. Mouth breathing 3-to-5-years-old children. Parrish Catia la Mar, Vargas. January–June 2008. *Rev. Med. Electrón.* [online]. 2010, vol.32, n.5, pp. 0-0. ISSN 1684-1824	Different study design
2	Rodríguez-Olivos, Lourdes Hilda Gabriela, Pamela Roxana Chacón-Uscamaita, Antony Germán Quinto-Argote, Graciela Pumahualcca, and Luis Fernando Pérez-Vargas. “Deleterious Oral Habits Related to Vertical, Transverse and Sagittal Dental Malocclusion in Pediatric Patients.” *BMC Oral Health* 22, no. 1 (March 23, 2022): 88. https://doi.org/10.1186/s12903-022-02122-4.	Different outcome
3	Serra-Negra, Junia Maria, Saul Martins Paiva, Lívia Bonfim Fulgêncio, Bertha Angelica Chavez, Carolina Freitas Lage, and Isabela Almeida Pordeus. “Environmental Factors, Sleep Duration, and Sleep Bruxism in Brazilian Schoolchildren: A Case-Control Study.” *Sleep Medicine* 15, no. 2 (February 1, 2014): 236–39. https://doi.org/10.1016/j.sleep.2013.08.797.	Different outcome
4	Lesinskiene, Sigita, Kamile Pociute, Asta Dervinyte-Bongarzoni, and Odeta Kinciniene. “Onychophagia as a Clinical Symptom: A Pilot Study of Physicians and Literature Review.” *Science Progress* 104, no. 4 (October 2021): 1–11. https://doi.org/10.1177/00368504211050288.	Different study design
5	Ellington, Erin. “Chronic Nail Biting in Youth.” *Journal of Psychosocial Nursing and Mental Health Services* 55, no. 2 (February 1, 2017): 23–26. https://doi.org/10.3928/02793695-20170210-03.	Different population
6	Costa, Julia Garcia, Genilza Silva Costa, Carolina Costa, Oswaldo de Vasconcellos Vilella, Claudia Trindade Mattos, and Adriana de Alcantara Cury-Saramago. “Clinical Recognition of Mouth Breathers by Orthodontists: A Preliminary Study.” *American Journal of Orthodontics and Dentofacial Orthopedics: Official Publication of the American Association of Orthodontists, Its Constituent Societies, and the American Board of Orthodontics* 152, no. 5 (November 2017): 646–53. https://doi.org/10.1016/j.ajodo.2017.03.025.	Different population
7	Carlsson, Gunnar E., Inger Egermark, and Tomas Magnusson. “Predictors of Bruxism, Other Oral Parafunctions, and Tooth Wear over a 20-Year Follow-up Period.” *Journal of Orofacial Pain* 17, no. 1 (2003): 50–57.	Different study design
8	Marceliano, Camila Rita Vicente, and Maria Beatriz Duarte Gavião. “Possible Sleep Bruxism and Biological Rhythm in School Children.” *Clinical Oral Investigations* 27, no. 6 (June 2023): 2979–92. https://doi.org/10.1007/s00784-023-04900-y.	Different outcome
9	Rauch, Angelika, Ina Nitschke, Sebastian Hahnel, Sophia Weber, Andreas Zenthöfer, and Oliver Schierz. “Prevalence of Temporomandibular Disorders and Bruxism in Seniors.” *Journal of Oral Rehabilitation* 50, no. 7 (2023): 531–36. https://doi.org/10.1111/joor.13450.	Different population
10	Tsitadze, T., S. Puturidze, T. Lomidze, V. Margvelashvili, and M. Kalandadze. “PREVALENCE AND RISK-FACTORS OF BRUXISM IN CHILDREN AND ADOLESCENT POPULATION AND ITS IMPACT ON QUЕALITY OF LIFE (REVIEW).” *Georgian Medical News*, no. 310 (January 2021): 36–39.	Different study design
11	Okawara, Aina, Yusuke Matsuyama, Miyu Yoshizawa Araki, Yuko Unnai Yasuda, Takuya Ogawa, Tsasan Tumurkhuu, Ganjargal Ganburged, Amarsaikhan Bazar, Takeo Fujiwara, and Keiji Moriyama. “Association between Child Abuse and Poor Oral Habits in Mongolian Adolescents.” *International Journal of Environmental Research and Public Health* 19, no. 17 (August 26, 2022): 10667. https://doi.org/10.3390/ijerph191710667.	Different outcome
12	Brandão de Almeida, André, Rita Salgado Rodrigues, Carina Simão, Raquel Pinto de Araújo, and Joana Figueiredo. “Prevalence of Sleep Bruxism Reported by Parents/Caregivers in a Portuguese Pediatric Dentistry Service: A Retrospective Study.” *International Journal of Environmental Research and Public Health* 19, no. 13 (June 25, 2022): 7823. https://doi.org/10.3390/ijerph19137823.	Different study design
13	Hsueh, Chang-Wei, and Chia-Wen Chen. “Prevalence of Nail Biting and Its Chronological Relationship with Tics in Child and Adolescent Outpatients with Tourette Syndrome: A Single-Centre, Retrospective Observational Study.” *BMJ Open* 12, no. 9 (September 15, 2022): e063874. https://doi.org/10.1136/bmjopen-2022-063874.	Different study design
14	Masuko, Alice Hatsue, Thais Rodrigues Villa, Marcia Pradella-Hallinan, Alexander Joseph Moszczynski, Deusvenir de Souza Carvalho, Sergio Tufik, Gilmar Fernandes do Prado, and Fernando Morgadinho Santos Coelho. “Prevalence of Bruxism in Children with Episodic Migraine--a Case-Control Study with Polysomnography.” *BMC Research Notes* 7 (May 14, 2014): 298. https://doi.org/10.1186/1756-0500-7-298.	Different study design
15	James P. Winebrake, Kartikey Grover, and Shari R. Lipner Pierre Halteh. “Pediatric Onychophagia: A Survey-Based Study of Prevalence, Etiologies, and Co-Morbidities | Request PDF.” *ResearchGate* 19 (December 9, 2024): 887–91. https://doi.org/10.1007/s40257-018-0386-1.	Different population
16	Quashie-Williams, R., O. O. daCosta, and M. C. Isiekwe. “Oral Habits, Prevalence and Effects on Occlusion of 4–15 Year Old School Children in Lagos, Nigeria.” *The Nigerian Postgraduate Medical Journal* 17, no. 2 (June 2010): 113–17.	Full text unavailable
17	Atsü, Saadet Sağlam, Sibel Güner, Nilgün Palulu, Ali Can Bulut, and Işın Kürkçüoğlu. “Oral Parafunctions, Personality Traits, Anxiety and Their Association with Signs and Symptoms of Temporomandibular Disorders in the Adolescents.” *African Health Sciences* 19, no. 1 (March 2019): 1801–10. https://doi.org/10.4314/ahs.v19i1.57.	Different population
18	University of Lund, Sweden, and Erik Larsson. “Artificial Sucking Habits: Etiology, Prevalence and Effect on Occlusion.” *International Journal of Orofacial Myology* 20, no. 1 (November 1, 1994): 10–21.	Different study design
19	Larsson, E., B. Ogaard, and R. Lindsten. “Dummy- and Finger-Sucking Habits in Young Swedish and Norwegian Children.” *Scandinavian Journal of Dental Research* 100, no. 5 (October 1992): 292–95. https://doi.org/10.1111/j.1600-0722.1992.tb01073.x.	Different outcome
20	Ngom, Papa Ibrahima, Falou Diagne, Joseph Samba Diouf, Allé Ndiaye, and Martine Hennequin. “[Prevalence and factors associated with non-nutritive sucking behavior. Cross sectional study among 5- to 6-year-old Senegalese children].” L′ Orthodontie Francaise 79, no. 2 (June 2008): 99–106. https://doi.org/10.1051/orthodfr:200803.	Full text unavailable
21	Graber, T. M. “Thumb- and Finger-Sucking.” *American Journal of Orthodontics* 45, no. 4 (April 1, 1959): 258–64. https://doi.org/10.1016/0002-9416(59)90084-3.	Full text unavailable
22	Guaba, K., G. Ashima, A. Tewari, and A. Utreja. “Prevalence of Malocclusion and Abnormal Oral Habits in North Indian Rural Children.” *Journal of the Indian Society of Pedodontics and Preventive Dentistry* 16, no. 1 (March 1998): 26–30.	Full text unavailable
23	Stefănescu, Iulia Marinela, Irina Zetu, and M. Rusu. “[News on prevalence of vicious habits in children].” *Revista Medico-Chirurgicala a Societatii De Medici Si Naturalisti Din Iasi* 115, no. 4 (2011): 1258–61.	Full text unavailable
24	Onyeaso, C. O., and E. O. Sote. “Prevalence of Oral Habits in 563 Nigerian Preschool Children Age 3–5 Years” *The Nigerian Postgraduate Medical Journal* 8, no. 4 (December 2001): 193–95.	Full text unavailable

## Appendix I Search Strategy

### PubMed search

Search date: 15/02/2024.

### Cochrane search

Date of search: 7/5/2014.

### ProQuest search

Date of search: 26/03/2024.

### Scopus search

Date of search: 25/03/2024.

### EBSCO-Metadata

Date of search: 07/05/2024.

### Epistemonikos Search

Date of search: 18/03/2024.

## Appendix III

Registered with PROSPERO, Centre for Reviews and Dissemination, University of York, with Registration number CRD42024511134.
